# The Story of the Insane from Year to Year

**Published:** 1894-05-19

**Authors:** 


### THE STORY OF THE INSANE FROM YEAR TO YEAR.

Seventh Series.

### THE BRISTOL ASYLUM.

On January 1st, 1893, there were 594 patients in residence here, and at the end of the year the numbers had risen to 616. There were 182 admissions, 70 recoveries, and 79 deaths, and these figures show that the percentage of recoveries was 38·46 and the percentage of deaths 12·97, the former being a little under the general average of asylums and the latter a little over it. Hereditary influence and intemperance in drink accounted for 62 of the admissions, or, to put it another way, one-third of the admissions had no business to exist at all. Thirty-nine of the deaths were caused by brain diseases, by far the most frequent of these being general paralysis of the insane; indeed, this cause appears so often that the high death-rate is thereby accounted for. Consumption was the cause in five only. There was one fatal case of enteric fever, and Dr. Benham tells us that the strictest inquiry was made into all the circumstances of the case, and it could not be traced to its origin. We have in these notices recorded our opinion that a disease clinically identical with enteric fever unaccountably appears now and then in asylums and shows no tendency to spread. The Visiting Committee say they "have much pleasure in expressing their high appreciation of the manner in which the medical superintendent and the officers and attendants have carried on the work of the asylum during the past year." The farm is credited with a balance of £73, but we see nothing charged for rent of land or interest on capital. £267 worth of vegetables were sent to the asylum, but at what price is not stated. 5,104 lb. of pork are charged at £132 10s., which is something like 6d. per pound, and he will be a lucky farmer that gets that price. If the average weekly cost per head were anywhere stated we failed to notice it.

### GLASGOW ROYAL ASYLUM.

It is very unfortunate for England that it possesses none of these Royal asylums. Our lunatic hospitals ought to be to some extent parallel institutions, receiving deserving cases at greatly reduced rates of payments; but most of our hospitals prefer to relieve the rich, and therefore the unfortunate lunatic of slender means too surely drifts towards our county asylums. The Glasgow Asylum contained 501 patients on the first day of the year 1893, and by the end of December the number had fallen to 490. Of this total 358 were private patients, and 132 were paupers. This pauper element is an ever-diminishing one, and the directors think that in a very few years the asylum will contain only those patients who are able to pay the whole or part of their maintenance. We are assured that the benevolent purpose of the asylum is never forgotten, and that many necessitous cases have been received at extremely moderate rates. This word "many" is a very elastic one, and we should like to see a table published with the report giving details of payments made by patients. The admissions during the year were 144, the recoveries were 53, and the deaths 47. The recoveries work out at 36·8 per cent. of the admissions, and the deaths at 9·4 on the average number resident. Of the year's admissions 23 were caused by drink, and 10 by hereditary influence. Half of the deaths were caused by brain diseases, 6 to heart disease, and 9 to consumption. Regarding the latter, Dr. Yellowlees says it has a twofold relation to insanity. In one class of cases the insanity is secondary to the lung mischief, and in the other the lung disease is secondary to the insanity. This may be so; but we are not sure that a "phthisical insanity" has yet been accepted by the bulk of physicians, and it must be remembered that both phthisis and insanity are common diseases, and this must be taken into account when speaking of their co-existence. The committee gave their "best thanks to Dr. Yellowlees and his medical assistants for the able and efficient manner in which they had discharged their onerous duties." The Commissioners reports are not given. The farm is credited with a balance of £366 on the right side, which in these times of unexampled agricultural depression is wonderfully satisfactory; but the farm accounts are too meagre to enable us to see how this has been obtained.

### THE LIMERICK DISTRICT ASYLUM.

On page 19 of this report it is stated that the limit of accommodation is 500 beds, and yet there were 533 patients in residence on January 1st, 1893, and at the close of the year the numbers had risen to 555, showing that considerable overcrowding existed. There were 130 admissions, 51 recoveries, and 46 deaths. Calculated in the usual way, these numbers yield a percentage of 39·2 recoveries and 8·3 deaths. As causes of insanity, hereditary influence heads the list and accounts for 22 of the year's admissions, while "previous attacks" is given as the cause in 16, and drink and religion are respectively responsible for 9 and 7. On analysing the deaths we learn that brain disease was the cause in 18, heart disease in 10, and consumption in 9. In Dr. O'Neill's ordinary report on the asylum there is nothing particularly deserving of notice, but he prints a special report on the alleged increase of insanity, and he arrives at the conclusion that there is an actual increase in this terrible disease. Although the population of Ireland is steadily diminishing, the number of the insane is steadily increasing, and the reason given is most likely the correct one, namely, that emigration calls away the young and strong, leaving the old and feeble to struggle on until the inevitable breakdown occurs. Dr. O'Neill shows that 20 per cent. of the insanity of Limerick is caused by hereditary tendency, and that the abuse of alcohol swells the asylum population. Now here are two perfectly preventable causes on which we have in these columns again and again enlarged on. Surely it is time that some action was taken to lessen these patent evils. The farm shows a balance of £455 in favour, but we see no prices placed opposite the produce sent to the asylum, so that the table is useless. The average cost per head per annum was £21 13s.

